# Polymorphism of dioctyl-terthiophene within thin films: The role of the first monolayer

**DOI:** 10.1016/j.cplett.2015.04.027

**Published:** 2015-06-16

**Authors:** Christoph Lercher, Christian Röthel, Otello Maria Roscioni, Yves Henri Geerts, Quan Shen, Christian Teichert, Roland Fischer, Günther Leising, Michele Sferrazza, Gabin Gbabode, Roland Resel

**Affiliations:** a Institut für Festkörperphysik, Technische Universität Graz, Petersgasse 16, 8010 Graz, Austria; b ams AG, Tobelbader Strasse 30, 8141 Unterpremstätten, Austria; c Dipartimento di Chimica Industriale, “Toso Montanari”, Università di Bologna, Viale del Risorgimento, 4, 40136 Bologna, Italy; d School of Chemistry, University of Southampton, Southampton SO17 1BJ, United Kingdom; e Laboratoire de Chimie des Polymères, Université libre de Bruxelles, Campus de la Plaine, 1050 Bruxelles, Belgium; f Institut für Physik, Montanuniversität Leoben, Franz Josef-Straße 18, 8700 Leoben, Austria; g Institut für Anorganische Chemie, Technische Universität Graz, Stremayrgasse 9, A-8010 Graz, Austria; h Département de Physique, Université libre de Bruxelles, Campus de la Plaine, 1050 Bruxelles, Belgium

## Abstract

The origins of specific polymorphic phases within thin films are still not well understood. The polymorphism of the molecule dioctyl-terthiophene is investigated during the presence of a silicon-oxide surface during the crystallisation process. It is found that a monolayer of molecules forms two-dimensional crystals on the surface. In the case of thicker films crystalline islands are formed, a comparison of the three polymorphic phases observed within thin films and the thermodynamically more stable single crystal phases reveals distinct differences which can be related to an adaption of the molecular packing with the flat surface of the substrate.

## Highlights

• We solved five different crystal structures of the molecule dioctyl-terthiophene.
• We show that specific surface induced phases appears in thin films.
• We could show that the first monolayer acts as nuclei for crystallisation.
• We showed that the molecular packing in the monolayer is the origin of polymorphism.

## Graphical abstract

**Figure:**
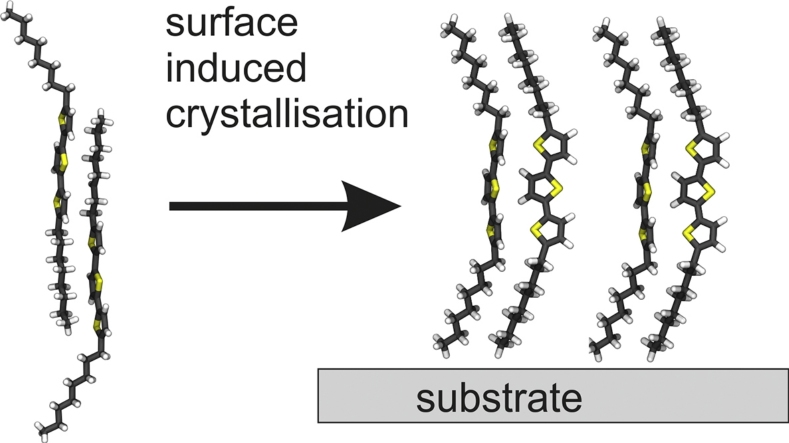

## 1 Introduction

The molecular organisation of thin films of organic semiconductors has been a topic of intense investigation in recent years [1]. The detailed knowledge of the crystal structure of a film in contact with a substrate (a dielectric for example) is of pivotal importance since it, in general, strongly influences the performance of organic electronic devices [2]. However, new polymorphic phases may appear due to the presence of a substrate surface during the crystallisation process [3], [4], [5]. This is an interesting fact, since the presence of different phases are important in several areas like organic electronics or pharmaceuticals. Such type of polymorphism is mainly observed in very thin films (<50 nm). For instance, pentacene shows a change of the triclinic unit cell as the thickness is decreased below a critical value of about 50 nm [6], [7]. Also, the presence of a substrate-induced phase in semiconducting liquid crystals has been observed for alkylcyanobiphenyl and for dioctylterthiophene (DOTT) [8], [9], [10]. Recently, we have observed a coexistence of two crystalline phases in thin films of DOTT, which we have called s- and b-phase [10]. Both phases are mediated by the substrate during thin film formation under conditions far from equilibrium [11]. A deeper understanding of the organisation of the film as the thickness is reduced to the monolayer level could be relevant for the understanding of the evolution of the different crystalline phases. Therefore, we performed an X-ray study, using both reflectivity and grazing incident diffraction techniques, on the organisation of DOTT films as the thickness is reduced to a single layer. We have clearly observed a new monolayer phase, different from the s- or b-phases, upon which, as the thickness of the film increases, both s- and b-phases develop. The crystal structures of these three substrate-induced phases are solved. Comparison with single crystal structures determined at room temperature and 100 K reveals differences in the conformation and packing of the molecules to the crystal structures formed within thin films.

## 2 Methods

The molecule DOTT shows three different smectic phases in between the isotropisation temperature (360 K) and the melting temperature of the crystalline state (335 K) [10], [12]. Samples were prepared by spin-coating onto thermally oxidised silicon wafers. The substrates were cleaned with acetone, isopropyl alcohol and in an ultrasonic bath of acetone for 15 min. The spinning program was 1000 rpm for 9 s, followed by 1500 rpm for 30 s. Solutions of DOTT (in toluene or tetrahydrofuran) were deposited with concentrations varying from around 0.1 to around 2 mg/ml (corresponding to a sub-monolayer and 10 layer-thick films, respectively). Films deposited at elevated temperature are prepared by heating both, the substrate and the solution. No significant differences between the samples prepared from toluene and tetrahydrofuran solutions are observed.

X-ray reflectivity (XRR) studies were performed in-house using a PANalytical Empyrean Reflectometer with a wavelength of *λ* = 0.154 nm. The experimental data were fitted with the software ‘X'Pert Reflectivity 1.3, PANalytical’ using Parratt's formalism, surface roughness was implemented by the model of Nevot and Croce [13], [14]. Grazing incidence X-ray diffraction (GIXD) was performed using the W1 Beamline at HASYLAB (Hamburg) with a wavelength of *λ* = 0.118 nm [15].

Atomic force microscopy (AFM) measurements were performed in tapping mode with an Asylum Research MFP-3D™ instrument equipped with an xyz closed-loop scanner. NSG30 cantilevers from NT-MDT have been used with spring constants of about 40 N/m. The applied force was minimised so that stable imaging was possible without squeezing the organic layers. The AFM topographic data were visualised and analysed using the Gwyddion SPM software v2.26. Optical microscopy was performed with an OLYMPUS BX51.

Molecular dynamics (MD) simulations were performed using LAMMPS in combination with the CHARMM General Force Field v. 2b7 [16], [17]. Density functional theory (DFT) calculations were carried out using the Vienna Ab Initio Simulation Package (VASP) using PAW potentials with an energy cut-off of 345 eV [18], [19], [20], [21]. The k-mesh was generated automatically by using a Monkhorst-Pack scheme.

Single crystals of DOTT were grown from toluene solution. Suitable crystals for X-ray structural analyses were selected and mounted on the tip of a glass fibre. Diffraction data were collected at 100 K and 296 K on a Bruker D8 Kappa diffractometer equipped with a SMART APEX II CCD detector. MoKα (*λ* = 0.071073 nm) radiation was used either from a sealed tube (1040724) or from a microsource (1040725). Solution of the crystal structures with direct methods and structural refinement was performed by SHELXS-97 [22]. The space group assignments and structural solutions were evaluated using PLATON [23]. The cif-files of all structures are given in the Supplementary Information.

## 3 Results and discussions

**Figure 1:**
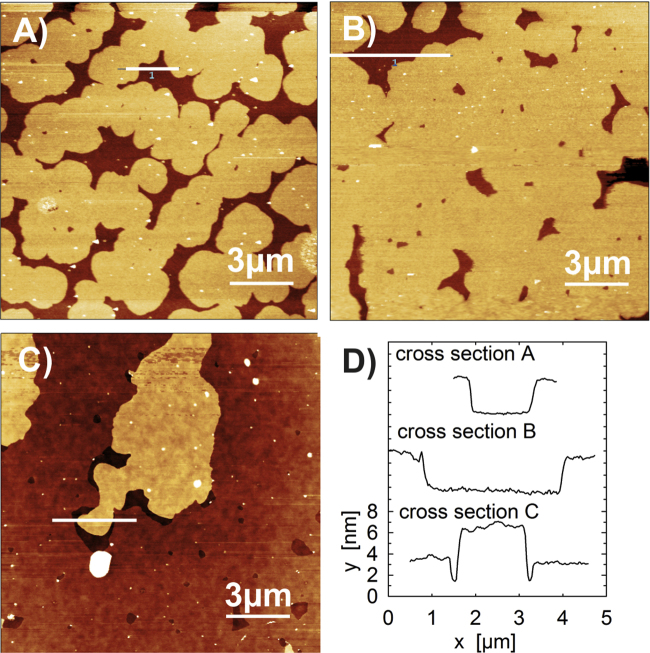
Atomic force microscopy images of three different thin films prepared by spin coating from different concentrations of the molecule DOTT in the solvent tetrahydrofuran (*z*-scale: 9 nm): 0.26 g/l (A), 0.33 g/l (B) and 0.43 g/l (C). For each micrograph a single line scan across the terraced morphology is given (D).

**Figure 2:**
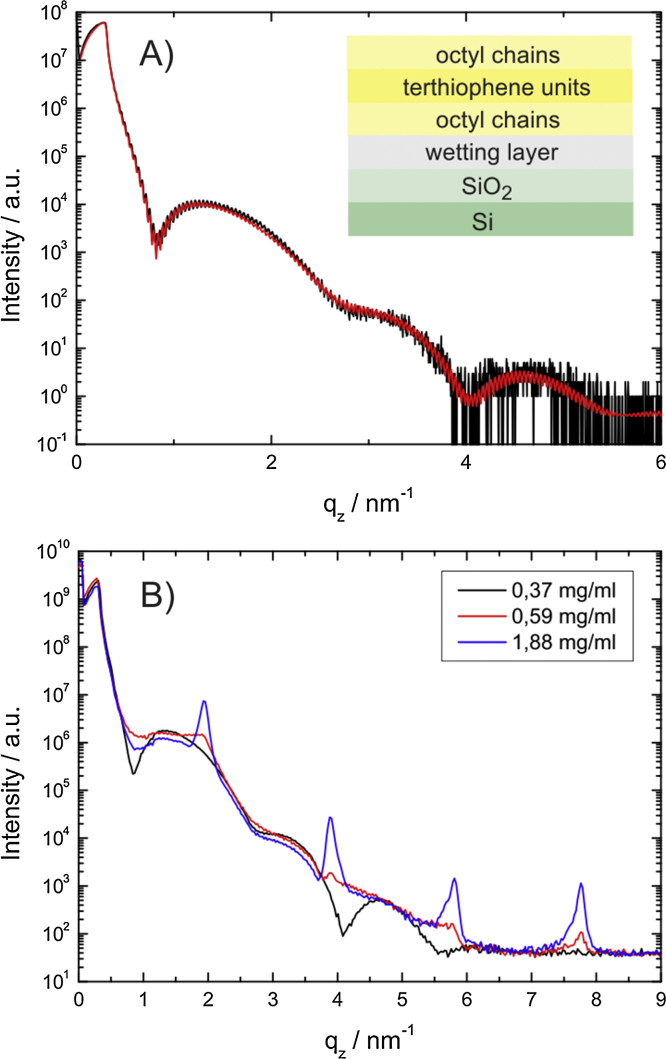
X-ray reflectivity curves of DOTT films prepared by spin coating. Experimental data (black line) of the film prepared from a concentration of 0.34 g/l and fitted curve (red line) based on the model plotted in the inset (A). Comparison of the experimental data for three different concentrations (B). (For interpretation of the references to colour in this figure legend, the reader is referred to the web version of this article.)

Figure 1 shows AFM images of films prepared from three different solution concentrations. At a concentration of 0.26 g/l, a submonolayer with a coverage of 80% is formed. The coverage increases to 91% at higher concentrations (0.33 g/l) and full coverage is reached at 0.37 g/l. Above this limit a second layer starts to form on top of the first closed monolayer, as shown in Figure 1c for a concentration of 0.43 g/l. Line scans reveal slight variations of the monolayer thickness between 2.9 nm (0.26 g/l) and 3.2 nm (0.33 g/l) which agree quite well with the length of an up-right standing DOTT molecule. A characteristic XRR profile of a monolayer is shown in Figure 2A. Pronounced Kiessig fringes are observed with maxima at 1.2 nm^−1^, 3.3 nm^−1^ and 4.6 nm^−1^, which are indicative of the presence of a monolayer. The fit of the reflectivity profile has been performed using a simple slab model including the silicon oxide layer on top of the bulk silicon substrate. The organic molecules themselves form three additional layers with different electron densities: a central layer of terthiophene units surrounded by two layers of densely packed octyl chains. The model used is drawn schematically in the inset of Figure 2A. The fit was improved if a ‘wetting layer’ of around 0.6 nm is included between the bottom octyl chains and the oxidised silicon substrate. This has been observed previously in other systems and can be related to the spin-coating procedure [24]. Figure 2A shows the excellent quality of the fit represented by the red line. The fit reveals a thickness of around 1.0 nm for the octyl chains and a thickness of 1.2 nm for the terthiophene layer. The total thickness of the monolayer from the XRR fit is 3.26 nm. The mass densities are between 0.6 g/cm^3^ (bottom) and 0.7 g/cm^3^ (top) for the octyl layers and 1.4 g/cm^3^ for the terthiophene layer, showing the increased electron density of the terthiophene core. Details of the X-ray reflectivity fitting procedure are given in the Supplementary Information.

**Figure 3:**
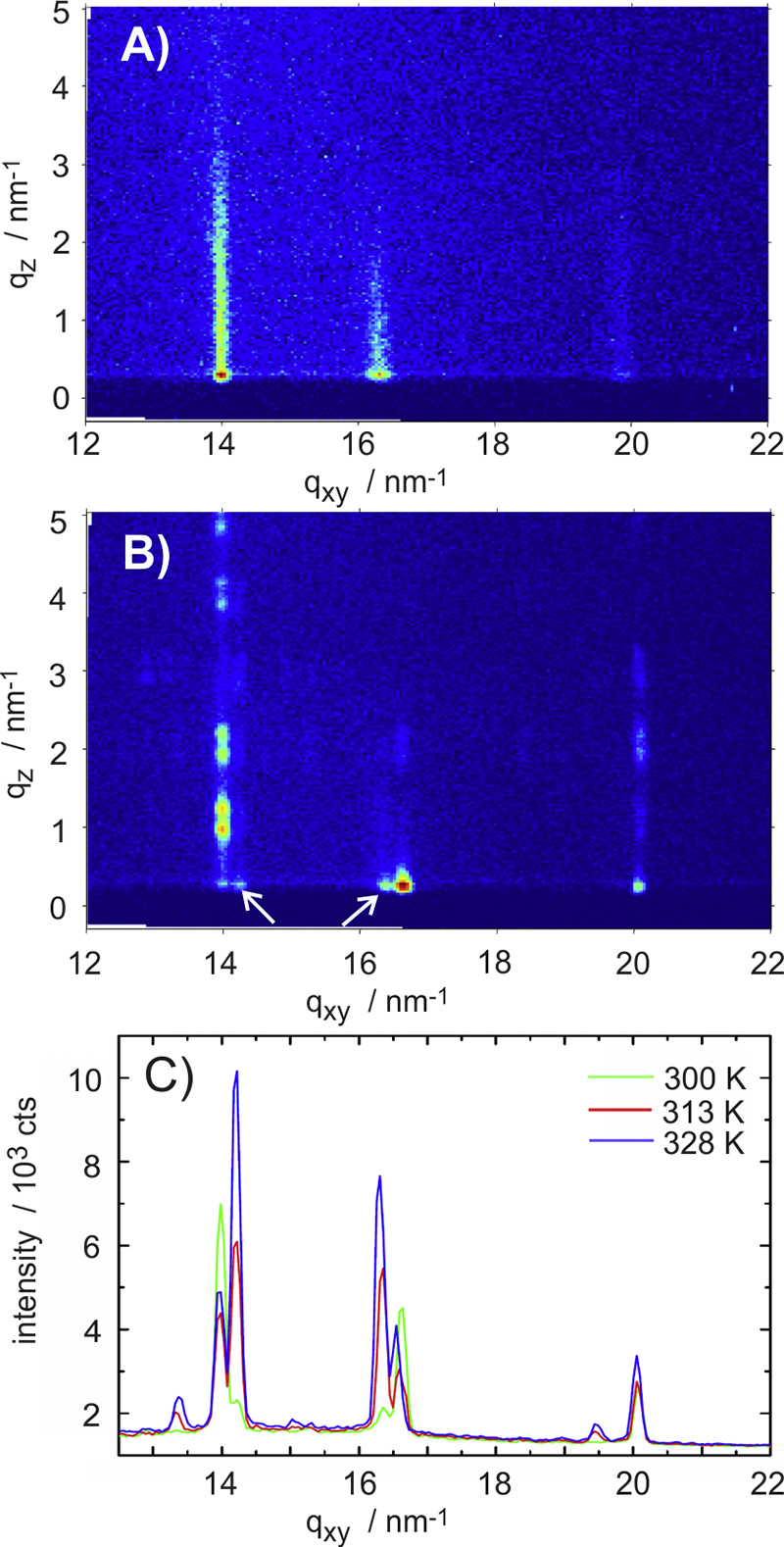
Reciprocal space maps of DOTT thin films prepared from different molecular concentrations: 0.44 g/l (A), 1.88 g/l (B). Integrated reciprocal space maps for films prepared at different temperatures using a concentration of 1.88 g/l (C).

To gain some insight into the internal structure of this monolayer, GIXD experiments were performed. The results are presented in Figure 3A as a reciprocal space map (*q*_xy_ versus *q*_z_). Three diffraction peaks are clearly visible at *q*_xy_ = 13.9 nm^−1^, 16.2 nm^−1^ and 19.8 nm^−1^ which extends in the *q*_z_ direction. The observation of Bragg rods instead of individual Bragg peaks reveals that the diffraction arises from two-dimensional crystals; this is clear evidence that the monolayer consists of crystalline domains. The *q*_xy_ positions of the Bragg rods are indexed to obtain the crystallographic unit cell, a rectangular lattice with *a* = 0.552 nm and *b* = 0.771 nm is found. This analysis reveals the presence of a new phase for the monolayer, different from the previously observed b- and s-phases.

**Figure 4:**
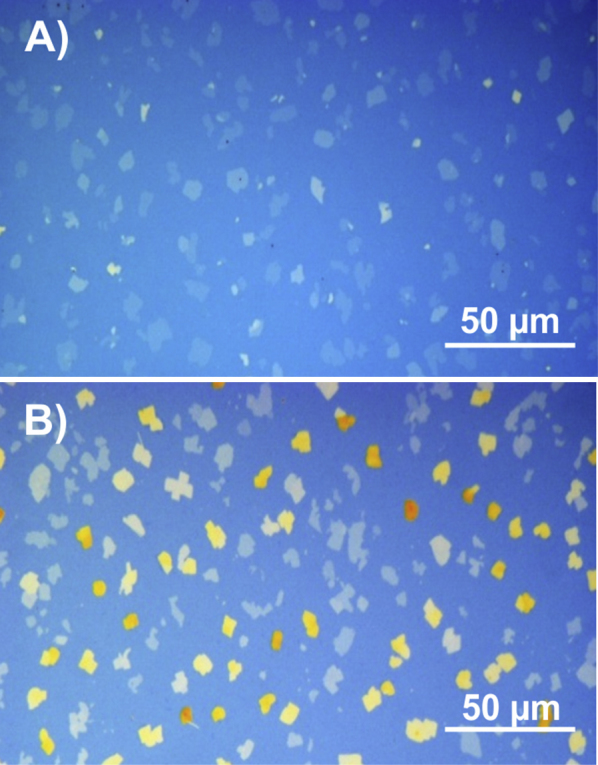
Optical microscopy images of DOTT thin films prepared from two different solutions with concentration 0.43 g/l (A) and 0.92 g/l (B).

The use of solutions with concentrations higher than 0.37 g/l yields samples with multilayer films. In the case of a concentration of 0.43 g/l (Figure 1C), the second layer has a height of 3.0 nm, as found from AFM line scans; this thickness is comparable with the thickness of the first monolayer. Optical microscopy studies reveal islands with a flake-like morphology, as shown in Figure 4A. The different optical contrast of the islands arises from the number of monolayers which are stacked upon each other. At higher concentrations, the flake-like character is well preserved since the crystallites grow preferentially in the vertical direction (Figure 4B). XRR profiles of the multilayer films are shown in Figure 2B together with the XRR profile of a closed monolayer. As the concentration is increased, the development of Bragg peaks is observed. A peak appears at 1.94 nm^−1^ together with its higher order reflections; this peak series is identified as *h* 0 0 of the b-phase. With increasing concentrations of the solution, the Bragg peaks become more intense and a shift of 0.02 nm^−1^ is observed in the position of the 1 0 0 peak. This can be related to interference between Bragg scattering and X-ray reflectivity [25]. A closer look to the XRR profiles reveals that the characteristic features of the monolayer are still present in the multilayer films. The maxima of the monolayer pattern at 1.2 nm^−1^, 3.3 nm^−1^ and 4.6 nm^−1^ are still present, but the minimum at 0.8 nm^−1^ is slightly smeared out. This experimental result reveals clearly that a monolayer is present in between the multilayer crystalline islands.

A GIXD pattern of the multilayer film is presented in Figure 3B, where well resolved Bragg peaks along vertical lines in the *q*_z_ direction are observed. The appearance of double peaks along *q*_z_ arises due to a dynamical scattering effect frequently observed for molecular crystals [26]. The GIXD pattern reveals the presence of three-dimensional crystallites with a strong preferred orientation in agreement with the specular X-ray diffraction measurements (Figure 2B). Even though the characteristic peak pattern of the b-phase is clearly observed, weak diffraction peaks of s-phase are also present in the diffraction pattern. These peaks are indexed as 0 1 1 (*q*_xy_ = 14.2 nm^−1^) and 0 0 2 (*q*_xy_ = 16.3 nm^−1^); both peaks are marked by white arrows in Figure 3B. Integrated GIXD patterns from thin films prepared at different temperatures are shown in Figure 3C. The integration is performed along *q*_z_ in the region 0–3 nm^−1^. At a preparation temperature of 328 K, predominantly the s-phase forms whilst at an intermediate temperature of 313 K both phases are present in approximately equal amount.

Table 1 gives the lattice constants of the crystallographic unit cells for the three polymorphs observed within thin films (monolayer phase, s-phase and b-phase). The lattice constants *a* and *b* are obtained by indexation of the GIXD pattern. The orthorhombic symmetry for the s- and b-phases allows the lattice constant *c* to be taken directly from specular X-ray diffraction measurements. In case of the monolayer phase, the total layer thickness is taken as the lattice constant *c* – as obtained from the XRR fit. Two molecules are packed within the unit cells which allows the calculation of the crystallographic density (Table 1). A combination of X-ray diffraction with MD simulations was used to solve the three crystallographic phases observed in thin films [27], [28]. The latter method uses a random initial guess for arrangement of two molecules in the crystallographic unit cell as a starting configuration for MD simulations. At this stage, a thermal annealing is carried out in order to overcome local energy minima. A selection of the structures with the lowest energy is subsequently minimised at the DFT level of theory in order to achieve a higher degree of accuracy.

**Table 1:** Lattice constants of three crystallographic phases of DOTT observed for thin films together with the calculated mass density (*ρ*), the type of molecular packing and the herringbone angle (Θ). The crystal structure was solved by a combination of grazing incidence X-ray diffraction and molecular dynamics simulations.

Phase	*a* nm^−1^	*b* nm^−1^	*c* nm^−1^	*ρ* g cm^−3^	Terthiophene packing	Θ °
Monolayer	0.552	0.771	3.26	1.13	Herringbone	55.3
s-Phase	0.543	0.771	3.32	1.13	Herringbone	56.4
b-Phase	0.559	0.756	3.24	1.15	Herringbone	52.6

Besides the three polymorphs observed within thin films, two further phases are found for single crystals grown from a toluene solution. Single crystal diffraction investigations were performed at two different temperatures (100 K and 296 K), the basic information on the crystal structure solution are listed in Table 2. A phase transition is observed from an orthorhombic phase (space group Pca2_1_) at *T* = 100 K to a monoclinic phase (P2_1_/c) at *T* = 296 K. Amongst the four known crystal structures at room temperature, the single crystal structure has the highest mass density. This is a clear indication that the single crystal structure is thermodynamically more stable than the three phases observed in thin films [29].

**Table 2:** Crystallographic information of two polymorphs of dioctylterthiophene observed at different temperatures. The crystal structure solution was performed by single crystal diffraction.

CCDC numbers	1040724	1040725
Formula	C_28_H_40_S_3_	C_28_H_40_S_3_
Formular weight	472.78	472.78
Temperature [K]	100(2)	296(2)
Crystal system	Orthorhombic	Monoclinic
Space group	Pca2_1_	P2_1_/c
*a* [nm]	6.3465(2)	0.55671(3)
*b* [nm]	0.55248(2)	6.3113(3)
*c* [nm]	2.95109(12)	0.78847(5)
*α* [°]	90	90
*β* [°]	90	107.604(3)
*γ* [°]	90	90
*V* [nm^3^]	10.3475(7)	2.6406(3)
*Z*	16	4
*ρ*_calc_ [g cm^−3^]	1.214	1.189
2*θ*_max_ [°]	56	53
Reflection measured/independent	127 554/26 774	25 377/5421
*R*(*F*) (>2*σ*/all reflections)	0.0539/0.0715	0.0580/0.0549
*wR*(*F*) (>2*σ*/all reflections)	0.1172/0.1243	0.1282/0.1244
Packing motif	Herringbone	Parallel stacking

**Figure 5:**
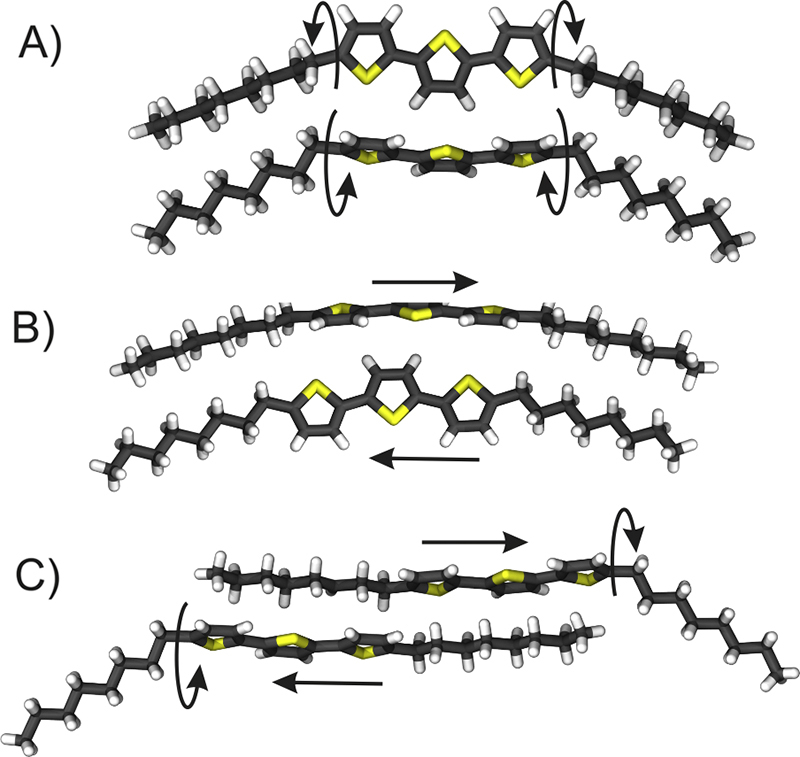
Packing of two neighbouring DOTT molecules as found in the different phases observed within thin films (A), within the single crystal phases at *T* = 100 K (B) and *T* = 296 K (C). The molecular packing is determined by a combined experimental/theoretical approach (A) and by single crystal X-ray diffraction (B, C). Arrows mark the differences in the conformation of the octyl side chains (A, C) and in the packing of the terthiophene units (B, C).

Figure 5 shows the different types of molecular packing found by crystal structure solution. A herringbone packing of the terthiophene units is observed for four of the polymorphic phases: for the three phases observed within thin films as well as for the single crystal phase at *T* = 100 K. The difference between the three thin film phases is evidenced by the herringbone angle Θ which is defined as the angle between the aromatic planes of two herringbone packed terthiophene units (Table 1). In the case of the single crystal structure the herringbone angle is 56°. Herringbone packing is frequently observed for elongated conjugated molecules, even for molecules with alkyl side chains at the terminal ends of the conjugated core [30], [31]. However, the single crystal structure at *T* = 296 K shows a parallel stacking of the aromatic units. This is observed quite rarely for rod-like conjugated molecules, yet some examples are reported in the literature [32].

Both methods of structure solution reveal a bent conformation of the central terthiophene units of the DOTT molecule as is clearly visible in all three cases in Figure 5. However, there is a fundamental difference in the conformation of the octyl side chains. Whilst for the single crystal phase at *T* = 100 K linearly extended chains are observed (Figure 5B), a defined rotation of the octyl chains relative to the terthiophene unit is found for the three thin film phases (Figure 5A). The rotation angle of about ±70° results from a twist of the first C—C single bond at the link between the terthiophene unit and the octyl chain (see arrows Figure 5A). Two features of this rotated conformation are interesting. First, a molecule with rotated side chains represents the equilibrium state of an isolated single DOTT molecule as obtained by combined MD and VASP calculations [33]. Second, the rotated conformation of the octyl chains allows a dense packing of the octyl side chains for both molecules. Interestingly, the single crystal structure at room temperature shows the twisted as well as the linear conformation of the octyl side chains within one molecule (Figure 5C).

There is also a distinct difference in the molecular packing between the thin film phases and the single crystal phases. Whilst for the thin film phases the terthiophene units are arranged side by side, in both single crystal phases some of the terthiophene units are shifted relative to each other. The direction of the shift is depicted by arrows in Figure 5B and C. A direct consequence of this packing is that the alkyl terminated ends of the molecules are not compatible with flat substrates. Conversely, the alkyl chains in the monolayer phase are arranged as to align their ending group with the support of the substrate which is a geometrical requirement for a substrate induced phase [5].

## 4 Conclusions

This study demonstrates the richness of the polymorphism exhibited by dioctylterthiophene (DOTT), especially when crystallised within thin films. Monolayers and multilayers of DOTT on a silicon oxide surface are investigated in terms of crystallographic structure and thin film morphology. Additionally, two crystal structures of DOTT are found for macroscopic single crystals. Layers with up-right standing molecules are formed on the substrate surface, with the total thickness of each layer being in between 3.24 nm and 3.32 nm. The first monolayer has a thickness of 3.26 nm and is composed of two-dimensional crystallites and is characterised by a rectangular lattice. This monolayer is always present, even when multilayer films are prepared, where crystalline islands together with the first monolayer are formed. Depending on the film preparation conditions, the islands can form two different phases: the b-phase (layer thickness 3.24 nm) is mainly present at 300 K whilst the s-phase (layer thickness 3.32 nm) becomes predominant at 328 K. The crystallographic structure of the three thin-film phases is determined by a combined experimental and theoretical approach. The conformation and the molecular packing in the first monolayer and in the two ‘island’ phases are similar, but quite different from the single crystal structures. Unlike the single crystal structures, the crystalline phases within thin films allows an adaption of the molecular packing with the highly planar substrate surface. The packing of the molecules within the first monolayer represents a template which defines the subsequent three-dimensional crystal growth at the substrate surface.
