# Supplementary material for: Polymorphism of dioctyl-terthiophene within thin films: The role of the first monolayer
Source: Chem Phys Lett. 2015 Jun 16;630:12–7. doi: 10.1016/j.cplett.2015.04.027 (PMC6472291; doi:10.1016/j.cplett.2015.04.027)
Supplement: Supplementary file 1 [file mmc1.pdf]

Polymorphism of dioctyl-terthiophene within thin films: the role of the first monolayer

Christoph Lercher,[a,b] Christian Röthel,[a] Otello Maria Roscioni,[c,d] Yves Henry Geerts,[e] Quan Shen,[f] Christian Teichert,[f] Roland Fischer,[g] Günther Leising,[a] Michele Sferrazza,[h] Gabin Gbabode,[h] and Roland Resel [a,*]

[a] *Institut für Festkörperphysik, Technische Universität Graz,Petersgasse 16, 8010 Graz, Austria*
[b] *ams AG, Tobelbader Strasse 30, 8141 Unterpremstaetten, Austria*
[c] *Dipartimento di Chimica Industriale, "Toso Montanari", Università di Bologna, Viale del Risorgimento, 4, 40136 Bologna, Italy*
[d] *School of Chemistry, University of Southampton, Southampton SO17 1BJ, United Kingdome*
[e] *Laboratoire de Chimie des Polymères, Université libre de Bruxelles, Campus de la Plaine 1050 Bruxelles, Belgium*
[f] *Institut für Physik, Montanuniversität Leoben, Franz Josef-Straße 18, 8700 Leoben, Austria*
[g] *Institut für Anorganische Chemie, Technische Universität Graz, Stremayrgasse 9, A-8010 Graz, Austria*
[h] *Département de Physique, Université libre de Bruxelles, Campus de la Plaine, 1050 Bruxelles, Belgium*

X-ray reflectivity fitting

The interpretation of x-ray reflectivity data relies strongly on fitting of the experimental data. The most elegant way is to determine the continuous change of the electron density vertical to the thin film surface [1]. A simpler approach – which is used in this work – uses a defined sequence of layers with variable layer thicknesses, electron densities and interface roughnesses [2]. Fitting of these three parameters for each individual layer can give a meaningful result. However, the quality of the fit depends strongly on the definition of a model which corresponds to thin film assembly.

In the present case a pre-experiment was required. An x-ray reflectivity measurement of the bare substrate – a thermally oxidised silicon wafer - was performed and fitted with a simple one-layer model assuming a single layer of amorphous silicon oxide on a silicon crystal. The obtained fit parameters of the layer and of the substrate are thickness, electron densities and interface roughness. These parameters were fixed for the subsequent experiment of the molecular monolayer. It is common practice that the electron density is converted to a mass density by assuming the chemical composition of the layer, here silicon dioxide.

The x-ray reflectivity measurement of the molecular monolayer deposited on thermally oxidised silicon was fitted in a first step by a four-layer model. The model was developed based on the pre-experiment of the bare substrate and results of Atomic Force Microscopy which reveals up-right standing molecules in the monolayer. The first layer represents the substrate consisting of silicon oxide on silicon. Three layers are assumed for the molecular monolayer, formed by three different electron densities. The carbon-hydrogen chains of the terminal ends (the octyl chains) are expected to have smaller densities than the central thiophene based part of the molecule. The starting values for the fit of the individual layers are chosen close to the expected values 1.0 nm and 1 g/cm^3 for the octyl

chains and 1.0 nm and 1.3 g/cm^3 for the central thiophene unit. The parameters for the substrate (silicon oxide on silicon) are fixed to 145.3 nm and 2.18 g/cm^3. The fit reveals an unexpected thickness of the octyl layer which is located close to the substrate surface. A value of 1.58 nm was obtained. Therefore, a five-layer model was assumed with one additional layer called "wetting layer" located in between the substrate surface and the octyl layer.

Table S1 gives the parameters of the final fit together with the upper and lower limit of the individual fit parameters. All parameters of the molecular layer – including the "wetting layer" were opened simultaneously during the last refinement process, while the parameters of the substrate were fixed. Table S2 gives the uncertainty of the fit parameters with upper and lower values of the error. Please note, that the given uncertainties are only numerical errors which result from the fitting procedure, experimental errors are not included. The experimental x-ray reflectivity curve (black line) and the fitted curve (red line) are depicted in Figure S1.

References:

[1] C. R. Hansen, T. J. Sørensen, M. Glyvradal, J. Larsen, S. H. Eisenhardt, T. Bjørnholm, M. M. Nielsen, R. Feidenhans'l, B. W. Laursen, Structure of the Buried Metal−Molecule Interface in Organic Thin Film Devices, Nano Lett. 9 (2009) 1052.

[2] O. Werzer, R. Resel, Model-Independent X‑ray Reflectivity Fitting for Structure Analysis of Poly(3-hexylthiophene) Films, Macromolecules 46 (2013) 3529.

Layer	density [g/cm³]	min/max [g/cm³]	thickness [nm]	min/max [nm]	roughness [nm]	min/max [nm]
octyl	0.72	0.5/1.2	0.97	0/1.5	0.35	0/1.5
thiophene	1.39	0/2.0	1.24	0/1.5	0.52	0/1.5
octyl	0.58	0.5/1.2	1.04	0/1.5	0.47	0/1.5
wetting	0.81	0.3/2.0	0.61	0/2.0	0.25	0/1.5
silicon oxide	2.18		145.3		0.38	0/1.5
silicon	2.328		infinite		0.20	

Table S1: Final fit parameters for the five-layer model for the mass density, thickness and roughness together with the lower limit (min) and the upper limit (max) of the fit parameter. Those values without limits are obtained by a pre-experiment and fixed during the fitting procedure.

Layer	density [g/cm³]	error min/max [g/cm³]	thickness [nm]	error min/max [nm]	roughness [nm]	error min/max [nm]
octyl	0.72	0.697/0.802	0.97	0.959/0.993	0.35	0.321/0.360
thiophene	1.39	1.350/1.418	1.24	1.228/1.255	0.52	0.495/0.613
octyl	0.58	0.563/0.667	1.04	1.028/1.046	0.47	0.396/0.521
wetting	0.81	0.744/0.833	0.61	0.603/0.620	0.25	0.231/0.327
silicon oxide	2.18		145.3		0.38	0.369/0.381
silicon	2.328		infinite		0.20	

Table S2: Numerical uncertainties of the fit parameters with upper and lower values of the error bar.

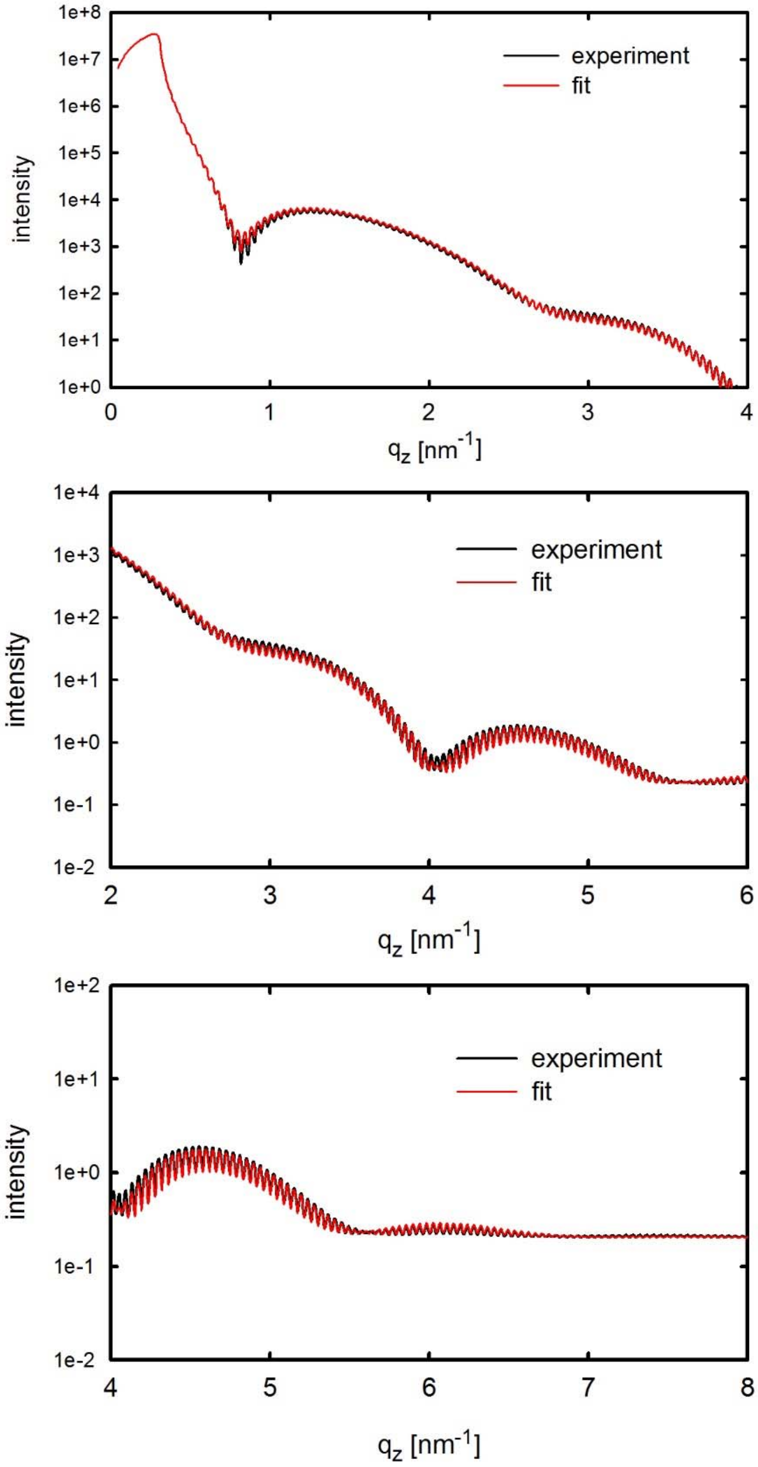

Figure S1: X-ray reflectivity of a monolayer prepared by spin coating from a tetrahydrofuran solution of the molecule dioctyl-terthiophene using a concentration of 0.34 g/l. The monolayer is prepared on a 145.3 nm thick thermally oxidised silicon wafer. The fit (red) of the experimental data (black) is performed by a five-layer model revealing mass density, thickness and roughness of each individual layer.
